# Omics Profiles of Non-transgenic Scion Grafted on Transgenic RdDM Rootstock

**DOI:** 10.14252/foodsafetyfscj.D-21-00012

**Published:** 2022-03-04

**Authors:** Hiroaki Kodama, Yukiko Umeyama, Taira Miyahara, Taichi Oguchi, Takashi Tsujimoto, Yoshihiro Ozeki, Takumi Ogawa, Yube Yamaguchi, Daisaku Ohta

**Affiliations:** 1 Graduate School of Horticulture, Chiba University, 1-33 Yayoi-cho, Inage-ku, Chiba 263-8522, Japan; 2 Faculty of Life and Environmental Sciences, University of Tsukuba, 1-1-1 Tennodai, Tsukuba, Ibaraki 305-8572, Japan; 3 Tsukuba Plant Innovation Research Center, University of Tsukuba, 1-1-1 Tennodai, Tsukuba, Ibaraki 305-8572, Japan; 4 Faculty of Engineering, Tokyo University of Agriculture and Technology, 2-24-16 Naka-cho, Koganei, Tokyo 184-8588, Japan; 5 Graduate School of Life and Environmental Sciences, Osaka Prefecture University, 1-1 Gakuen-cho, Naka-ku, Sakai, Osaka 599-8531, Japan

**Keywords:** Keyword genetically modified (GM) plants, grafting, new plant breeding technology (NPBT), omics analysis, RNA-directed DNA methylation (RdDM), siRNA

## Abstract

Grafting of commercial varieties onto transgenic stress-tolerant rootstocks is attractive approach, because fruit from the non-transgenic plant body does not contain foreign genes. RNA silencing can modulate gene expression and protect host plants from viruses and insects, and small RNAs (sRNAs), key molecules of RNA silencing, can move systemically. Here, to evaluate the safety of foods obtained from sRNA-recipient plant bodies, we investigated the effects of rootstock-derived sRNAs involved in mediating RNA-directed DNA methylation (RdDM) on non-transgenic scions. We used tobacco rootstocks showing RdDM against the cauliflower mosaic virus (CaMV) 35S promoter. When scions harboring CaMV 35S promoter sequence were grafted onto RdDM-inducing rootstocks, we found that RdDM-inducing sRNAs were only weakly transported from the rootstocks to the scion, and we observed a low level of DNA methylation of the CaMV 35S promoter in the scion. Next, wild-type (WT) tobacco scions were grafted onto RdDM-inducing rootstocks (designated NT) or WT rootstocks (designated NN), and scion leaves were subjected to multi-omics analyses. Our transcriptomic analysis detected 55 differentially expressed genes between the NT and NN samples. A principal component analysis of proteome profiles showed no significant differences. In the positive and negative modes of LC-ESI-MS and GC-EI-MS analyses, we found a large overlap between the metabolomic clusters of the NT and NN samples. In contrast, the negative mode of a LC-ESI-MS analysis showed separation of clusters of NT and NN metabolites, and we detected 6 peak groups that significantly differed. In conclusion, we found that grafting onto RdDM-inducing rootstocks caused a low-level transmission of sRNAs, resulting in limited DNA methylation in the scion. However, the causal relationships between sRNA transmission and the very slight changes in the transcriptomic and metabolomic profiles of the scions remains unclear. The safety assessment points for grafting with RdDM rootstocks are discussed.

## 1. Introduction

Plant breeding frequently involves hybridization between two plants. Typically, one parental plant with superior agricultural traits for abiotic and/or biotic stress, such as drought, salinity, abnormal temperature tolerance, and resistances against plant pests and pathogens such as viruses, fungi and insects, is crossed with another parental plant that is less tolerant but possesses superior genomic traits of features linked to consumer preference, such as good nutrition, taste and yield. To combine these traits into a single plant cultivar or variety, numerous and repeated rounds of hybridizing and backcrossing, which involves tedious labor in the field, is required. In addition, this breeding technique is limited to only fertile and hybridizable plant species, cultivars and varieties.

Grafting is one technique that can help overcome the hybridizing barrier, since grafting can be implemented between different cultivars or even, in some cases, between unrelated plant species^1^^,^^2^^)^. Tough plants, such as wild plant species that possess tolerance for environmental stresses and/or virus and pathogen attacks, but are not suitable for commercial consumption, can be used as rootstocks, and scions of different plant species can be grafted onto the rootstock. For example, in Japanese seedling markets, grafted seedlings from the Solanaceae species such as eggplants, tomatoes and bell peppers, as well as seedlings from the Cucurbitaceae, including cucumber, cantaloupe and watermelon, are widely distributed. Many species of fruit trees including peach, apple, and persimmon are grafted onto a rootstock to confer improved resistance against environmental stresses, thereby enabling rapid propagation of elite cultivars by farmers. The advantage of grafting is that, once when one elite rootstock cultivar is developed, the superior traits of the rootstock can be applied to many and wide varieties of scion plants and be distributed into markets within a couple of years. When one genetically modified (GM) plant is once developed to be suitable for rootstocks harboring transgenes for virus, pathogen and insect resistance and abiotic stress tolerance, it is expected that the GM seedlings are used for grafting with the scions of many non-GM varieties and cultivars, moreover beyond species, having superior traits for foods but fragile against these biotic and abiotic stresses during cultivation on farms to overcome these harms. In this case, the transgenes are not introduced into the scion part and the fruits ripening on the scions should be non-GM foods.

Grafting has been used throughout the academic study of plant signaling pathways; i.e., the study of the regulation of development by signaling substances such as RNAs, proteins, oligopeptides, and plant growth regulators, because grafted plants permit the movement of signaling molecules from the signal source parts of the plant body to the signal-receiving parts. Thus, grafting permits scientists to alter and modulate the development of target parts via long-distance signaling transmission through the grafted junction^3^^)^. One classic example whereby the regulatory mechanisms responsible for plant morphology were elucidated by grafting techniques is the original proof of the mobility of florigen, which was shown over 80 years ago; florigen has now been identified as a product of the *FT* gene^4^^,^^5^^,^^6^^)^. Another mobile signal is mediated by 21- to 24- nucleotide (nt)-long small RNAs (sRNAs) such as small interfering RNA (siRNAs) and micro RNAs (miRNAs). These sRNAs are incorporated into Argonaute (AGO) proteins and guide RNA silencing processes. These include post-transcriptional gene silencing (PTGS), which is associated with sRNA-mediated degradation of RNA molecules, as well as transcriptional gene silencing (TGS) by RNA-directed DNA methylation (RdDM)^7^^)^. The first evidence of the long-distance transmission of PTGS was observed in tobacco plants transformed with nitrate reductase (NR) or nitrite reductase (NiR) genes^8^^)^. When these transgenes were introduced into tobacco plants, two phenotypes were observed: plants showing successful overexpression of the transgene and those showing sense transgene-induced PTGS (S-PTGS). When scions of the overexpressors were grafted onto S-PTGS rootstocks, the phenotype of scions changed from overexpression of the transgene to PTGS. The mechanisms responsible for cell-to-cell movement of sRNAs via plasmodesmata and long-distance sRNA transmission via phloem have been studied using tobacco and Arabidopsis^9^^)^. Finally, sRNAs have also been known to play important roles in the regulation of plant genes, in the modulation of plant development, in the allocation of nutrients, and in the virus resistance^10^^,^^11^^,^^12^^,^^13^^,^^14^^)^.

RNA silencing has been applied during production of high oleate GM soybean, virus-resistant papaya, low-lignin alfalfa, insect-resistant maize, non-browning apple and potato, etc.^15^^)^. In addition to these GM crops, the combined application of RNA silencing and grafting has also been attempted in numerous crops. For example, in one study a non-GM sweet cherry scion was grafted onto a transgenic cherry rootstock showing siRNA-mediated virus resistance^16^^)^. The resultant non-GM scion thereby acquired enhanced virus resistance. In potato, grafting-mediated induction of DNA methylation may also be an attractive prospect. DNA methylation can be induced in the potato rootstock by grafting with tobacco scions producing siRNAs targeting the endogenous promoter sequences of potato^17^^)^. Because potato is typically propagated vegetatively and DNA methylation is generally maintained in vegetative tissue, DNA methylation induced by grafting was maintained in the progeny tubers without the tobacco scions. In addition, several other studies have shown that siRNAs and miRNAs can transmit to grafted plants, in which the source of siRNA is the rootstock, and the target is the scion to cause repression of target genes in the scion^16^^,^^17^^,^^18^^,^^19^^,^^20^^,^^21^^,^^22^^)^.

The safety of food products obtained using new plant breeding technologies (NPBTs) is also an important concern. NPBTs include the genome editing, oligo-directed mutagenesis, cisgenesis and transgenesis, RdDM, and grafting^23^^)^. In a previous paper, we showed that a transgenic protein (β-glucuronidase) expressed in GM tomato rootstock did not cause unintended effects on transcriptomic, proteomic, or metabolomic traits affecting food safety in the non-GM scion^24^^)^. Here, to evaluate the risk of grafting techniques involving epigenetic regulation, we investigated the effects of RdDM-inducing tobacco rootstock on a non-GM scion (i.e., as a model testing the long-distance movement of siRNAs). The cauliflower mosaic virus (CaMV) 35S promoter and derivative promoters such as El2 are often used for constitutive overexpression of downstream genes. When a hairpin construct harboring a partial sequence of the CaMV 35S promoter in an inverted repeat manner is introduced into tobacco plants, a resultant transgenic locus (end2) can induce RdDM of target sequences^25^^)^. Here, we used this RdDM-inducing GM tobacco line as a model rootstock to elucidate the unintended effects on the transcriptomic, proteomic and metabolomic traits of a non-GM scion.

## 2. Materials and Methods

### 2.1 Preparation of Grafted Plants Consisting of Tobacco Showing RdDM as a Rootstock and GM Tobacco Harboring the Target Promoter Sequences as a Scion

**Fig. 1.**
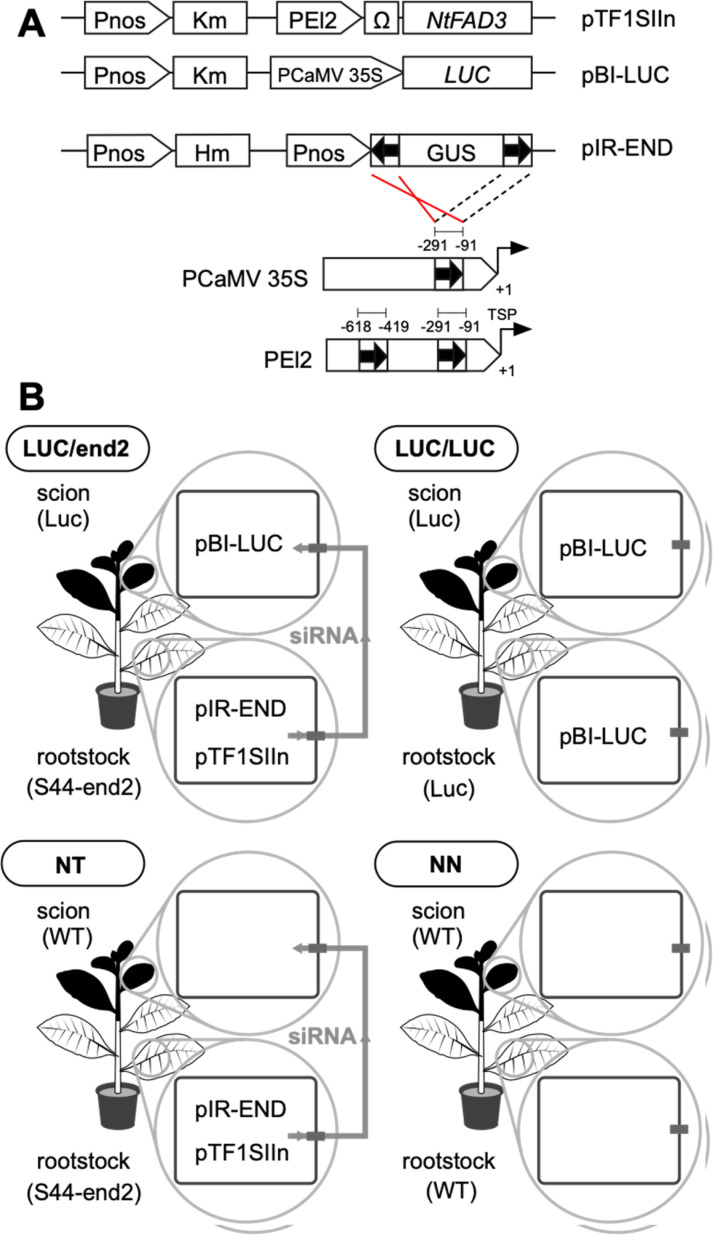
Schematic diagrams of binary vectors for the production of transgenic plants (A) and scion and rootstock combinations (B). Pnos, nopaline synthase promoter; Km, neomycin phosphotransferase II gene; PEL2, El2 promoter; Ω, 5′-leader sequence of tobacco mosaic virus; *NtFAD3*, cDNA encoding tobacco endoplasmic reticulum ω-3 fatty acid desaturase; PCaMV 35S, CaMV 35S promoter, *LUC*, firefly luciferase gene; Hm, hygromycin phosphotransferase gene; GUS, β-glucuronidase gene; LUC/end2, grafted plants between the LUC scion and the S44-end2 rootstock; LUC/LUC, grafted plants between the LUC scion and rootstock; NT, grafted plants between non-GM, WT scion (N) and transgenic S44-end2 rootstock (T); NN, grafted plants between WT scion and rootstock.

An RdDM-inducing construct, pIR-END, produces siRNAs targeting two regions, from −618 to −419 and from −219 to −91 from the transcription start point (TSP) of the El2 promoter^25^^)^. Because the El2 promoter contains two tandemly repeated enhancer regions of the CaMV 35S promoter, pIR-END also targets a similar enhancer region (−219 to −91 from TSP) of the CaMV 35S promoter (**Fig. 1**). This RdDM construct was introduced into S44 tobacco plants that were homozygous for the *NtFAD3* transgene, pTF1SIIn^26^^)^. *NtFAD3* encodes an endoplasmic reticulum ω-3 fatty acid desaturase, and the NtFAD3 protein catalyzes the desaturation from linoleate (18:2) to linolenate (18:3). pTF1SIIn contains *NtFAD3* cDNA under the control of the El2 promoter. The homozygous S44 line shows the S-PTGS phenotype; namely a leaf fatty acid composition with extremely low 18:3 content. After introduction of pIR-END, the resultant S44-end2 line shows an overexpressor phenotype of the *NtFAD3* transgene^25^^)^. This conversion of the S44 phenotype is explained as follows: S-PTGS in the parental S44 line was induced by the strong transcription of the *NtFAD3* transgene, and this high rate of transcription was attenuated by the methylation of the El2 promoter after introduction of pIR-END. Since the S-PTGS was not induced by such low levels of transcription, the residual low level of transcription of the *NtFAD3* transgene in the S44-end2 line caused successful production of the NtFAD3 protein which caused efficient conversion of 18:2 to 18:3. Therefore, the phenotype of the S44-end2 plants showed the overexpressor phenotype, i.e., high 18:3 content in leaf tissue. The offspring of the S44-end2 line showing the high 18:3 phenotype was used as rootstocks. The leaf 18:3 content was determined as previously described^27^^)^.

When the grafting-induced DNA methylation and rootstock-derived siRNAs were determined in the scion, a transgenic tobacco line harboring pBI-LUC (designated a LUC line) was used as the scion (Fig. 1). pBI-LUC harbors a firefly luciferase gene under the control of the CaMV 35S promoter^28^^)^.

Grafting was performed by a cleft graft^8^^)^. Approximately 5-week-old, aseptically growing S44-end2 and LUC seedlings were cut 4 cm above the medium surface. The LUC scion was grafted onto a S44-end2 or LUC rootstock and was subsequently cultured in aseptic conditions (Fig. 1B, LUC/end2 and LUC/LUC). Grafted plants were transferred to soil 2 weeks after grafting (WAG). Leaves and petioles near the graft junction were collected at 4 WAG for bisulfite sequencing. Petioles near the graft junction were collected at 4 WAG for next generation sequencing (NGS) of siRNAs.

### 2.2 Bisulfite Sequencing

Total DNA was prepared from the leaves and petioles of LUC scions grafted onto the S44-end2 rootstocks according to the method of Edwards et al^29^^)^. Total DNA was then further purified using a DNA purification column (Favorgen Biotech Corp., Taiwan), and was quantified using a Qubit DNA Assay Kit (Thermo Fisher Scientific, MA, USA). Bisulfite treatment was performed using a Fast Bisulfite Conversion Kit (Abcam, Cambridge, UK). The target region of pIR-END and the 5′ region of luciferase gene were amplified. The resulting PCR products were cloned into pGEM-T Easy Vector (Promega, WI, USA), and randomly picked clones were sequenced. The primers used for amplification of the DNA fragment from the target region of the siRNA to the 5′ region of luciferase gene were as follows: El2-end2-upper-Fw, 5′-AGAAGAYYAAAGGGYTATTGAGA-3′ and end2-luc-Rv, 5′-CCATCCTCTARARRATARAAR-3′.

### 2.3 NGS of sRNA

Total RNA was prepared using Sepazol-RNA I Super G reagent (Nakarai Tesque, Kyoto, Japan). The sequence profile of the sRNA was obtained as a service by DNAFORM (Kanagawa, Japan) and registered as BioProject ID: PRJDB11010, Experiment ID: DRX300543-44. The data analysis tools used to obtain the expression levels of genes of interest (Fragments Per Kilobase of gene/transcript per Million mapped reads: FPKM) were as follows: cutadapt (v2.10), FastQC (0.11.9), STAR (v2.7.3a), featureCounts (v2.0.1), DESeq2 (v1.20.0), MBCluster.Seq (v1.0), and the DNAFORM original script. The draft genome sequence of *Nicotiana tabacum* used for mapping was the Nitab-v4.5_genome (i.e., the dataset of Edwards 2017, solgenomics.net).

### 2.4 Preparation of Grafted Tobacco Plants Consisting of Tobacco Showing RdDM as a Rootstock and Non-GM Tobacco as a Scion

Approximately 5-week-old, aseptically growing S44-end2 and wild-type (WT) seedlings were cut 4 cm above the medium surface. The WT scion was grafted onto S44-end2 or WT rootstocks and was subsequently cultured in aseptic conditions (**Fig. 1B**, NT and NN). The grafted plants with a S44-end2 rootstock and a WT scion were designated NT, and those with both WT scion and rootstock were designated NN. All grafted plants were transferred to soil at 2 WAG. During the first 7 d after planting, grafted plants were covered with a glass beaker. Plant tissues were collected at 6 WAG as follows: mature 12th leaves from the graft junction were collected for transcriptomic analysis and 8th leaves from the graft junction were collected for metabolomic analysis. For the proteome analysis, total protein was prepared using three mature leaves corresponding to the 9th to 14th positions from the graft junction. The collected samples were frozen with liquid nitrogen and stored at −80°C until use. The scion leaves of three independently grafted plants were subjected to the omics analyses to provide biological replicates.

### 2.5 Transcriptomic Analysis of the Scion Leaves of Grafted Tobacco Plants

Total RNA from frozen-stocked tobacco leaves was extracted using the FavoPrep Plant Total RNA Mini Kit (Favorgen Biotech Corp., Taiwan). The outsourcing service of Eurofins Genomics (Tokyo, Japan) constructed the RNA library and obtained mRNA sequencing data. The mRNA was purified as poly(A)^+^ RNA, and paired-end 101-base sequencing data were generated using a HiSeq 4000 platform (Illumina Inc., San Diego, CA). The mRNA-seq dataset (BioProject ID: PRJDB11010, Experiment ID: DRX300537-42) contained a total of 44.3 million reads. Adapter sequences were then trimmed, and low-quality reads containing poly-N and/or being less than 50 bp in length were discarded using fastp (v0.20.1).

Although the draft genome sequence of *Nicotiana tabacum* was registered, this data has not been updated since 2017. Thus, we generated a new *de novo* assembly using Trinity (v2.11.0) for transcriptome analysis. Benchmarking Universal Single-Copy Orthologs (BUSCO, v2.0.1) were obtained to calculate assembly statistics for evaluation. The obtained contigs were first subjected to quality control by removing redundancy using trinity script files (align_and_estimate_abundance.pl and filter_low_expr_transcripts.pl) and CD-HIT (v4.8.1) and then were used for alignment by bowtie2 (v2.4.2). RNA-seq by expectation-maximization (RSEM) (v1.3.3) was used to calculate gene expression levels, which were represented by transcripts per million (TPM) values. Prediction of encoded peptides was annotated using BLASTp searches of the Uniprot database by Transdecoder (v5.5.0). Hierarchical cluster analysis and identification of differentially expressed genes (DEGs) were performed by stats (v3.6.1), edgeR (v3.28.1), and R package (v3.6.1). Gene ontology (GO) enrichment analysis was performed by Database for Annotation, Visualization and Integrated Discovery (DAVID, v6.8).

### 2.6 Proteomic Analysis of the Scion Leaves of Grafted Tobacco Plants

A hundred milligrams of pulverized tobacco leaves were suspended in 500 μL of CellLytic P extraction buffer (Sigma-Aldrich Japan Co., Tokyo, Japan) and then vortexed for 1 min followed by centrifugation at 10,000 rpm for 10 min. After filtration through a membrane filter (0.45 μm; Merck Millipore, Billerica, MA, USA), 100 μL of 5 mM iodoacetamide was added to the filtrate and incubated in the dark for 10 min. After subsequent centrifugation for 30 min, 10 units of trypsin was added and incubated overnight at 37°C. After filtration through a membrane filter, the filtrate was passed through a C_18_ spin column (washed with 1% trifluoroacetic acid (TFA) in H_2_O and eluted with 1% TFA in 80% acetonitrile), then dried under vacuum and stored at −80°C. The dried peptides were then dissolved in a small aliquot of water and introduced to ultra-high performance liquid chromatography (UHPLC)-electrospray ionization (ESI)-tandem mass spectrometry (MS/MS) by a Q Exactive hybrid quadruple-orbitrap mass spectrometer with an Ultimate 3000 RS LC nano system (Thermo Fisher Scientific, Waltham, MA, USA). This system was equipped with an ACQUITY BEH C18 column (particle size 1.7 mm, 100 × 2.1 mm, Waters, Milford, MA, USA) and was connected to a Vanguard BEH C18 pre-column (particle size 1.7 mm, 5.0 × 2.1 mm, Waters). The eluting solvents were A: 0.1% aqueous formic acid solution and B: 0.1% formic acid−acetonitrile solution (both LC-MS grade, Kanto Chemical Co., Ltd.), and the elution conditions were 5%B−95%B (0−40 min) with a flow rate of 0.05 mL/min gradient. The analyzing condition for mass spectrometry (MS) was as follows: ionization: positive and negative mode; capillary temperature: 320°C; vaporization temperature: 300°C; desolvation gas: nitrogen; spray voltage: 4.0 KV; cone voltage: 35.0 V; collision voltage: 30.0 V; and measurement range: *m/z* 150−2000. MS/MS was performed using the all-ion fragmentation method. The resolution was set to 60,000 (at 400 *m/z*), the AGC target was 1e6 and maximum injection time was set to 120 msec. The MS/MS resolution was set to 17,500, with an isolation window of 2 *m/z*, an underfill ratio of 1.3%, an AGC target of 5e5, and maximum injection time of 100 msec. Dynamic exclusion was set to 120 msec. For calibration, we used a Thermo Scientific Pierce LTQ Velos ESI Positive Ion Calibration Solution and a Thermo Scientific Pierce ESI Negative Ion Calibration Solution (Thermo Fisher Scientific). All data obtained from UHPLC-MS in both the positive- and negative-ion modes were processed using Progenesis QI data analysis software (Nonlinear Dynamics, Newcastle upon Tyne, UK). This was used for peak picking, alignment, and normalization to produce peak intensities for retention time and *m/z* data pairs. The ranges of the automatic peak picking assays were between 2 and 40 min. The resultant data matrices were imported into SIMCA version 14.0 (Umetrics, Umeå, Sweden) for further multivariate statistical analysis with Pareto scaling.

### 2.7 Metabolomic Analysis of the Scion Leaves of Grafted Tobacco Plants

The metabolome was analyzed using high performance liquid chromatography (LC)-ESI-MS and gas chromatography (GC)-electron ionization (EI)-MS following a previously reported protocol^24^^)^.

For LC-ESI-MS analysis, metabolites were extracted using the method of Iijima et al^30^^)^ with some modifications. Each lyophilized sample was grounded into powder in liquid nitrogen. Thirty mg of ground sample was mixed with 900 µl of 75% methanol containing reserpine (20 µg/ml) as an internal control. After homogenization using a Mixer Mill MM 400 (Retsch, Haan, Germany) with a zirconia bead at 30 Hz for 2 min, the homogenate was centrifuged at 12,000 × *g* for 10 min at 4°C. The extraction was repeated twice, and the supernatants were combined in a new microcentrifuge tube. The supernatant was filtered through a 0.2-µm PTFE membrane (Millex-LG; Merck Millipore, Billerica, MA). Non-targeted metabolite analysis was carried out by LC–ESI-MS using a LCMS–8040 system (Shimadzu Corp., Kyoto, Japan) as described previously^24^^)^. Mass spectra within the *m*/*z* range of 100–1000 were obtained by Q3 scan mode with positive/negative polarity switching. Using ProteoWizard’s MSConvertGUI software^31^^)^, a set of LC–ESI-MS raw data files was converted to mzXML file format. The mzXML files were uploaded to XCMS Online ver. 3.7.0^32^^)^ to process the dataset. The mass data obtained between 2−12 min were analyzed by the XCMS using a provided parameter set #11025 with the “matchFilter” feature detection method.

The extraction, phase separation, derivatization, and GC-EI-MS analysis steps for the tobacco leaf samples were performed as described previously^33^^)^. In brief, a Micromass GCT Premier Mass Spectrometer (Waters, Milford, MA) connected to an Agilent 6890 Gas Chromatograph (Agilent Technologies) and an autosampler (PAL GC-xt; CTC Analytics, Zwingen, Switzerland) was used to acquire data. MassLynx 4.0 software (Waters) was used to control the GC-EI-MS system. The derivatized samples and an *n*-alkane mixture (from C8 to C40) were analyzed independently in the same GC-EI-MS run. MetAlign^34^^)^ was used to automatically correct the baseline and to align all extracted mass peaks across all samples. AIoutput2^35^^)^ was used to deconvolute ion peaks, to convert retention times to retention indices, and to annotate peaks using the in-house mass spectral library.

Principal component analysis (PCA) was performed using the web-based free software MetaboAnalyst 4.0^36^^)^. The data scaling used for PCA was automatic (i.e., auto scaling), which is mean-centered and divided by the standard deviation of each variable.

## 3. Results

### 3.1 Effects of Rootstocks Showing RdDM on DNA Methylation Levels in siRNA-Recipient Scions

**Fig. 2.**
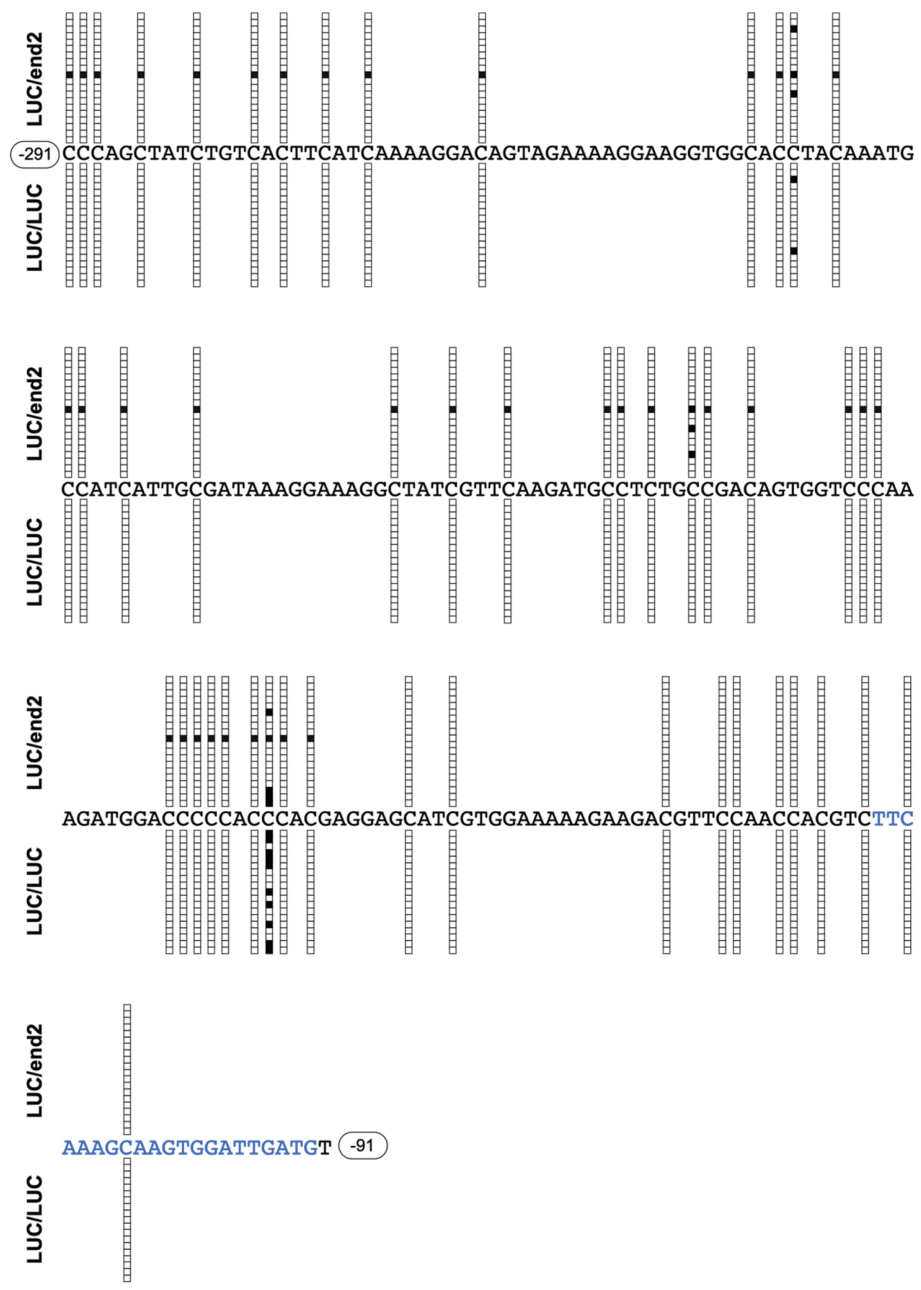
Cytosine methylation status in the target sequences of a RdDM construct, pIR-END. DNA methylation status of the leaf tissues of LUC scions grafted onto S44-end2 or LUC rootstocks. Shown are the CaMV 35 promoter sequences corresponding to the target region of pIR-END (–291 to –91 from TSP). White and black boxes indicate the unmethylated and methylated cytosine residues in PCR clones prepared from bisulfite-treated DNA samples. A single 21-nt-long siRNA was detected in two independently prepared siRNA libraries and is indicated by blue letters.

S44-end2 plants produce siRNAs that induce the methylation in the enhancer region of the CaMV 35S promoter^25^^)^. To determine whether siRNAs produced in the S44-end2 rootstocks were able to induce RdDM in the scion, we used LUC plants as scions. This GM scion harbors a transgene consisting of CaMV 35S promoter and luciferase cDNA. LUC scions were grafted onto S44-end2 rootstocks (designated LUC/end2) and LUC rootstocks (designated LUC/LUC), and we then examined the methylation of target region located in the CaMV 35S promoter (**Fig. 1**). Leaves at 12 to 17 mm above the grafting junction were harvested at 4 WAG. Total DNA purified from scion samples was subjected to bisulfite treatment. The target region (−291 to −91 from TSP) was amplified and cloned (**Fig. 2**). One clone of the LUC/end2 sample was methylated in the RdDM target region. Methylation was preferentially observed in the target region of the pIR-END construct, and the flanking upstream promoter region (−341 to −292 from TSP) was not methylated (**Table S1**). None of clones of the LUC/LUC sample showed methylation. In contrast, the cytosine residues of the target region and its upstream flanking region were highly methylated in the leaves of the S44-end2 rootstock (**Table S1**). In the latter case, the direct repeat structure of the enhancer region of the El2 promoter (**Fig. 1A**) may affect methylation at the upstream flanking region.

**Fig. S1.**
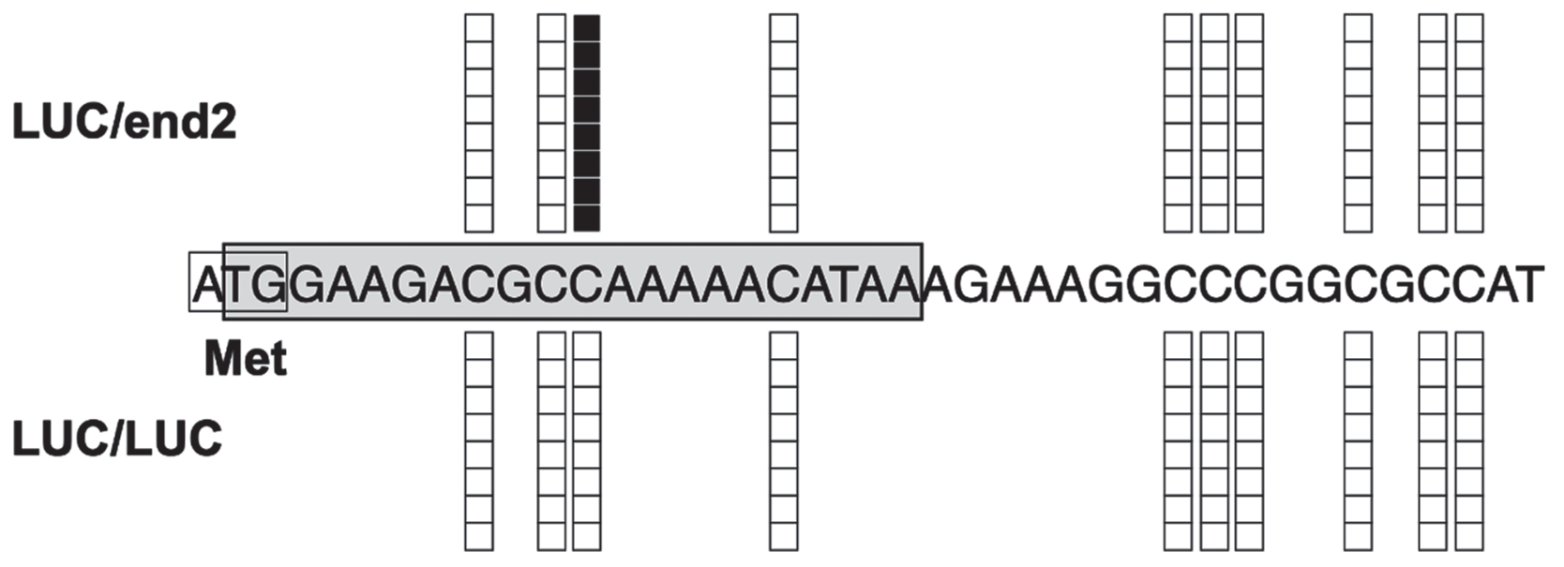
DNA methylation in the luciferase gene preferentially observed in the LUC/end2 grafted plants. DNA methylation status of leaf tissue of LUC scions grafted onto S44-end2 or LUC rootstocks. The 5′ cDNA region encoding luciferase is shown with the translation start codon. The white and black boxes indicate the unmethylated and methylated cytosine residues in eight independent PCR products from the bisulfite-treated DNA samples. We detected two copies of one 21-nt siRNA molecule harboring the complementary sequences of the 5′ terminal region of the luciferase gene from the scion of a LUC/end2 grafted plant (indicated by a gray box).

Next, we determined the composition of the sRNAs present via NGS. Only one 21-nt-long siRNA harboring the target sequences was detected in two independently prepared sRNA libraries (Fig. 2). These results indicate that transmission of siRNA from the rootstock to scion occurred at very low —nearly negligible— levels, and only a small number of cells in the scion received these siRNAs and showed the methylation by RdDM. Interestingly, one cytosine residue near the translation initiation codon of the luciferase gene was found to be highly methylated in the scion of LUC/end2 plants but not in the corresponding scion of LUC/LUC plants (**Fig. S1**), indicating that grafting onto the RdDM-showing rootstocks could also cause unintended DNA methylation. Furthermore, we detected one 21-nt siRNA molecule harboring complementary sequences of the 5′ terminal region of the luciferase gene (**Fig. S1**). This siRNA was detected as two copies in one of the two prepared sRNA libraries. Because the 24-nt-long siRNA is thought to direct RdDM^7^^)^, the involvement of the 21-nt-long siRNAs (Fig. 2 and **Fig. S1)** in DNA methylation in the scion is obscure. In addition, the luciferase activity in the scion of LUC/end2 plants was almost the same as in LUC/LUC plants (data not shown). Therefore, the DNA methylation status of the scion and siRNAs transmitted from the rootstock did not remarkably affect the expression of the luciferase transgene in the scion portion of LUC/end2 grafted plants.

### 3.2 Phenotype of Tobacco Plants Grafted onto the Rootstocks Showing RdDM

**Fig. 3.**
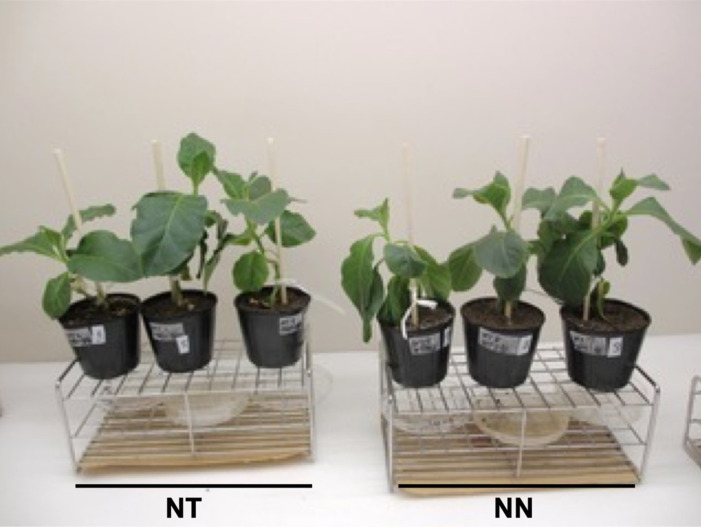
Phenotypes of grafted tobacco plants. NT designates the grafted plants with S44-end2 tobacco plant as rootstock and non-GM wild tobacco as scion. NN designates the grafted plants with non-GM tobacco as both rootstock and scion. Source plants for grafting, seedlings of GM and non-GM plants, were grown 5 weeks after germination and then cut and grafted. Grafted plants were further grown for 6 weeks.

Our previous experiment showed unremarkable differences in growth and phenotype between S44-end2 and non-GM wild plants^25^^)^. The growth pattern of grafted plants that used S44-end2 as a rootstock and WT as a scion (designated NT) was almost identical to the pattern of grafted plants that used both WT scions and rootstocks (designated NN). The phenotypic differences between NT and NN plants were also unremarkable (**Fig. 3**).

### 3.3 Transcriptomic Analysis of Scion Leaves Derived from NN and NT Grafted Plants

After quality filtering and RNA-seq read cleaning, we obtained approximately 43.4 million sequence reads, from which 118,007 contigs were assembled with an N50 of 1,662 bp. From a total of 1,440 BUSCO groups searched using the embryophyta_odb9 database, our contig sample showed that 1,249 (86.7%) were categorized as complete BUSCOs. To discard any redundancies, we ran trinity scripts and CD-HIT: this yielded a total of 35,804 high-quality transcripts, of which 32,797 were annotated based on Uniprot database.

**Fig. 4.**
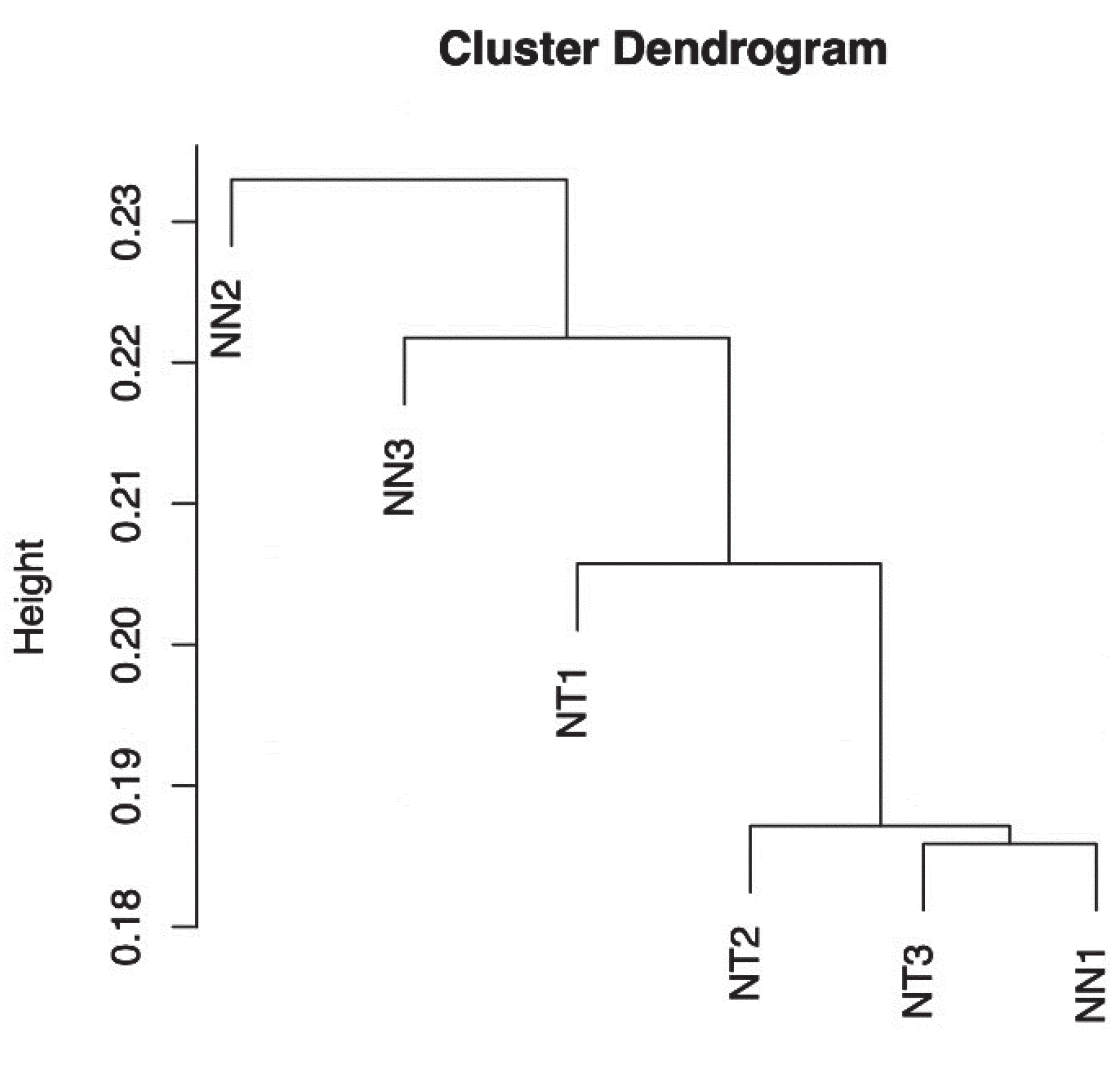
Hierarchical cluster tree of genes expressed by three grafted tobacco plants with non-GM tobacco as both scion and rootstock (NN1–3) and three grafted plants with non-GM tobacco as a scion and transgenic tobacco rootstock showing RdDM (NT1–3). Dendrogram generated from 35,804 genes expressed at least in one of the six samples.

Next, the 43.4 million RNA-seq reads, sourced from three scion samples each of NN and NT plants, were mapped against the 35,804 transcripts. Using this procedure, we identified a total of 35,639 genes expressed with TPM > 1, in at least one of the six samples. Hierarchical cluster analysis results indicated that the expression profiles were not divided into two discrete NN and NT groups (Fig. 4).

We then compared the transcriptomes of DEGs found in the three biological replicates of the NN and NT scions. This permitted the identification of 55 DEGs (P_FDR_ < 0.05), of which 25 genes were more highly expressed in NT than in NN scions, while the others were downregulated in NT scions (**Table S2**). GO enrichment analysis showed three genes that were categorized as “response to wounding”, all three of which were upregulated in the NT group. No other DEGs were clustered in specific molecular functions or biological processes.

Several reports showed that the promoter activity was repressed by an RdDM construct harboring a 200- or 261-bp-long target sequences but was not by the RdDM construct harboring 123-bp-long target sequences^37^^,^^38^^,^^39^^)^. These results suggest that effective size of the target region for RdDM is more than 200 bp in length. When the target sequences of pIR-END (−291 to −91 of CaMV 35S promoter) was subjected to BLASTn searches (i.e., showing similarity > 90% against 200-nt-long query sequences) of the Ntab4.5 tobacco genome assembly, the tobacco genomic sequences did not show significant similarity to the query sequences. Therefore, the possibility in which downregulated DEGs in the NT scion was due to methylation caused by siRNAs transmitted from the S44-end2 rootstock is low. In addition, the detected siRNA molecules described in Fig. 2 and **Fig. S1** do not show significant similarity to the tobacco genomic sequences. When mismatches with one or two nucleotides were included in the siRNA-target searches, these two siRNA sequences did not show any similarity to the tobacco genomic region. Therefore, the differential expression of the above DEGs between NT and NN samples would not be induced by the siRNA molecules detected in this study (Fig. 2 and **Fig. S1**). From these points, the off-target effects by the RdDM construct used in this study are likely to be negligible.

### 3.4 Proteomic Analysis to Scion Leaves Derived from NN and NT Grafted Plants

**Fig. 5.**
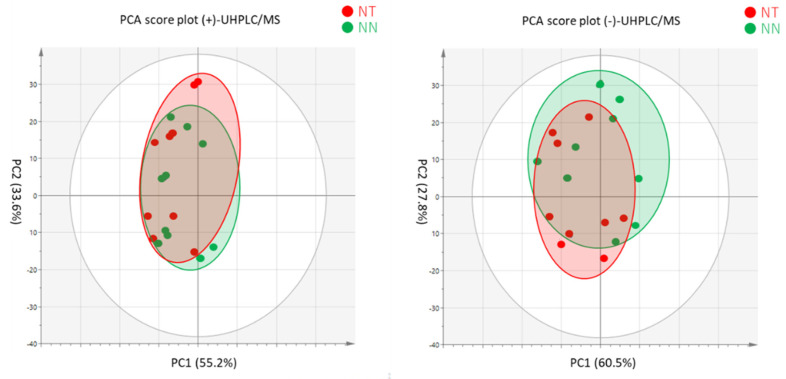
PCA score plot of data from NN and NT scion leaves for proteomic analysis performing using UHPLC-MS. a) ESI-(+)-UHPLC/MS; b) ESI-(–)-UHPLC/MS.

In the UPLC-MS analysis, 3,207 distinct peaks were detected, of which 1,384 peaks were annotated as tobacco peptides. Because most of them did not show the significant differences between the NT and NN samples, we carried out the multivariate analysis using the UHPLC-MS data. The PCA results are illustrated in a two-dimensional score plots. On these plots, the first and second principal components (PCs) are the two axes, which permits visual inspection of group differences between NN and NT scion leaf samples (**Fig. 5**). These PCA score plots showed no clear differences (i.e., cluster separation) between the NN and NT samples in both (+)- and (−)-UHPLC/MS measurements. Next, we performed a discriminant analysis by orthogonal partial least squares regression (OPLS-DA) for both groups. The explanatory value (*R^2^Y*) of the objective variable was as low as 0.821 for (+)-UHPLC/MS and 0.799 for (−)-UHPLC/MS, while the predictive value (*Q^2^Y*) was 0.621 for (+)-UHPLC/MS and 0.540 for (−)-UHPLC/MS. Thus, we were not able to construct a good discriminant model using these two groups, which suggests that there is no significant difference between them. Taken together, these results suggest that the expression of RdDM-inducing siRNAs in transgenic tobacco rootstocks did not have a significant effect on the translated products of non-GM tobacco scions.

### 3.5 Metabolomic Analysis of Scion Leaves Derived from NN and NT Grafted Plants

Metabolomic profiles were obtained using analytical data from non-targeted metabolomic profiling by LC-ESI-MS and GC-EI-MS. For MS chromatographic analyses, each metabolite should be independently eluted according to its unique physicochemical properties and should be distinguished as a unique ion signal. However, in general, each ion peak (including molecular ions, adduct ions, and fragment ions) usually overlaps with neighboring peaks to varying degrees. Thus, raw data from mass chromatograms must be processed to retrieve information from individual ion peaks. In this study, we resolved individual ion peaks by peak picking and peak deconvolution using several different software packages, each of which is suitable for a particular analytical platform (for details, see Materials and Methods section). These data processing procedures were archived based on *m/z* tolerance, retention time tolerance, and peak intensity correlation. Each of the clarified ion peaks (which we call “peak group” in this study) represents the signal from a single metabolite. In each peak group, an ion peak with the highest ion intensity was defined as the “representative ion peak”; this peak intensity value was then used for metabolic profile comparisons among samples.

**Fig. 6.**
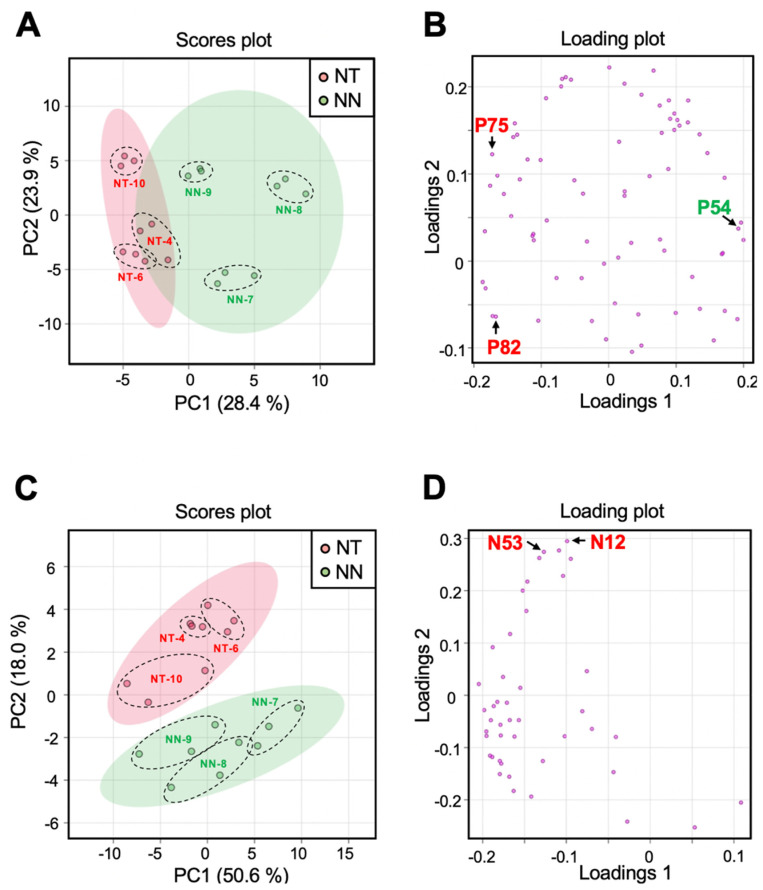
Comparison of metabolite composition in leaves from grafted tobacco plants. PCA score plots of the metabolic profiling data set obtained by LC-ESI-MS positive ion mode (A and B) and negative ion mode (C and D). Left panels (A and C) represent the score plot for PC1 vs. PC2. Each plot represents an individual analytical sample. Percentage values in parentheses are the respective contribution ratios. Right panels (B and D) represent the factor loading plots for PC1 vs. PC2. Each plot represents an individual ion selected from each peak group. Numbers besides the plots represent their Peak group ID. P54, P75, P82, N12 and N53 indicate the metabolites that showed more than two-fold difference between NT and NN lines with P_FDR_ < 0.05.

Mature leaves from the scion parts of NN and NT plants were subjected to LC-ESI-MS and GC-EI-MS analyses. During the LC–ESI-MS analysis, we detected 44 and 75 peak groups by ESI positive and negative ion modes, respectively. To compare the metabolic profiles, we performed a PCA on the metabolite data matrices obtained by the positive and negative ion modes. Two-dimensional PCA score plot graphs were generated with PC1 and PC2 as main axes (Fig. 6). For the positive ion mode results, the metabolomic clusters of the NT and NN samples were found to be partially overlapping (Fig. 6A). The contribution ratio of PC1 was 28.4% and that of PC2 was 23.9%. The PC1 axis likely differentiated NT clusters from NN clusters. On the PC1 axis, NT-4, NT-6, and NT-10 plants had slightly lower PC scores relative to the others. However, the PC1 scores of NN-9 was closer to those of NT plants. In terms of individual NN plants, the plots of NN-7, NN-8, and NN-9 were not tightly clustered on the two-dimensional PCA score plot. This result indicated that each NN plant had a unique metabolomic profile that might be attributable to individual variability. In the negative-ion mode, we observed separation of the NT and NN clusters (Fig. 6C). The contribution of PC1 was 50.6% and that of PC2 was 18.0%. Here, the PC2 axis likely differentiated the NT and NN clusters. With respect to the PC2 axis, NT-4 and NT-6 plants were found to have slightly higher PC scores than the others. On the other hand, NT-10 had a score closer to those of NN plants.

Next, 74 peak groups clarified in GC-EI-MS analysis were used as a data for a PCA to compare the metabolite profiles between NT and NN plants. The contribution ratio of PC1 was 31.4%, and that of PC2 was 22.2%. Here, no cluster separation was observed between NT and NN plants in the PC1−PC2 two-dimensional PCA score plots (data not shown).

**Fig. S2.**
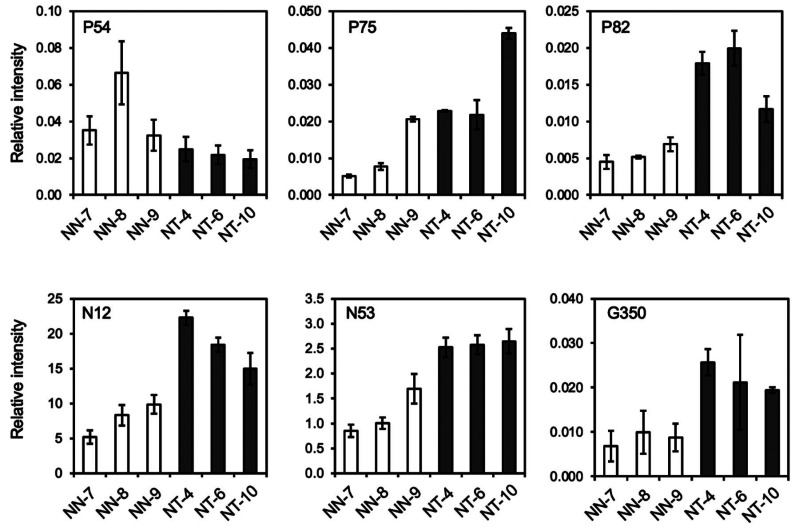
Relative levels of 6 metabolite signals that showed more than twofold difference between NT and NN lines with P_FDR_ < 0.05. Peak group IDs are shown on the top of the panels. Scions of three independent NN plants and three independent NT plants were analyzed. Values indicate average ± standard deviation (n = 3, technical replicates).

From the comparison of intensities of each peak group obtained by LC-ESI-MS and GC-EI-MS, we found that the relative levels of six peak groups significantly differed between NT and NN plants (Fig. 6B and D, and **Table S3**). Of these, P75, P82, N12, N53 (detected in the LC-ESI-MS analysis), and G350 (detected in the GC-EI-MS analysis) were present at higher levels in NT, whereas P54 (detected in the LC-ESI-MS analysis) was present at a lower level. To obtain structural information, we conducted LC-MS/MS analysis on the representative ions of P54, P75, P82, N12 and N53, and performed a public mass spectrum library search using the resulting MS/MS spectra as query. We were not able to retrieve plausible results to explain the molecular identities of these metabolites (data not shown). Similarly, G350 was not clarified through the comparison of mass spectrum and retention index values between G350 and the compounds in our in-house reference compound library. No compounds showed hits with identification scores of 0.700 or higher. The relative levels of the six peak groups in each grafted plant are shown in **Fig. S2**. All six peak groups were detected in both NT and NN plants. Further studies are necessary to clarify whether the differences in the level of accumulation of P54, P75, P82, N12, N53, and G350 were illustrative of within-individual variability or represented a metabolic response to the grafting of the non-GM scions on the GM rootstocks.

## 4. Discussion

The spreading of PTGS from the rootstock to the scion (or vice versa) in grafted plants was first observed in tobacco plants transformed with NR and NiR genes, in which the PTGS phenotype was spread into scions from a PTGS-showing rootstock^8^^)^. When local silencing was induced in green fluorescent protein-expressing tobacco plants, silencing spread throughout the plants, with the exception of the shoot and root apical meristems^40^^)^. This systemic silencing signal was demonstrated to be caused by sRNAs by an elegant grafting experiment using Arabidopsis silencing-related mutants^41^^)^. In our previous study, silencing induced by a hairpin construct harboring *NtFAD3* cDNA was spread into scions harboring the *NtFAD3* sense transgene^42^^)^. These observations demonstrated that sRNAs inducing PTGS move in the root to shoot direction. Although the systemic spreading of PTGS was clearly observed, several studies have shown that mobile sRNAs almost exclusively move from shoots to roots^41^^,^^43^^)^. For example, when RdDM-inducing scions were grafted onto non-GM potato rootstocks, efficient RdDM was established in the potato tubers^17^^)^. In fact, induction of RdDM by mobile sRNAs in the shoot to root direction has been observed in the grafted plants, but induction of DNA methylation in the scion grafted onto the RdDM-inducing rootstocks is not yet well understood^44^^)^. In this report, siRNAs targeting the enhancer region of the CaMV 35S promoter were detected at quite low levels in the scion, and only limited DNA methylation of the CaMV 35S promoter occurred in the scion after grafting onto rootstocks showing RdDM. Although these reports mentioned above showed that sRNA transmission from rootstock to scion is limited relative to transmission from scion to rootstock, siRNA transmission may be modulated by scion condition. For example, efficient transport of siRNAs from rootstock to scion was observed in the non-GM tomato scions grafted onto GM tomato rootstocks that had been modified to promote RNA silencing of a gene encoding plastid-localized ω-3 fatty acid desaturase^45^^)^. In this case, scion leaves had been removed before grafting, which should promote the transmission of substances from the rootstock to the scion^3^^)^. Thus, sRNA transmission beyond the graft junction is affected in response to scion condition, which controls the flow of substances in the phloem.

RNA silencing includes two different mechanisms: PTGS and TGS. The spreading of PTGS has been observed from rootstock to scion even though siRNA movement was limited. The spreading of PTGS is often associated with the synthesis of secondary siRNAs. The primary siRNAs produced in the rootstock are then transported into the scion, where they then guide the AGO1-mediated cleavage of complimentary RNA molecules. The resulting 5′-truncated and/or 3′-truncated RNA fragments are often converted into double-stranded RNA (dsRNA). These newly produced dsRNAs are further processed into siRNAs, known as secondary siRNAs^46^^)^. Notably, the generation of secondary siRNAs has been observed in scions that had been grafted onto rootstocks showing PTGS^42^^)^. Because secondary siRNAs are produced from the flanking regions of the sequences that are complementary to primary siRNAs, secondary siRNA production is associated with the broadened target region of PTGS in the scion. In contrast, our results show that the promoter regions methylated by RdDM in the scion had not broadened, and that DNA methylation of the flanking upstream and downstream promoter regions in the scion did not change after grafting (Table S1). In this respect, grafting of a WT scion onto a rootstock showing RdDM should preferentially induce DNA methylation at predictable target regions in the scion. One notable change was unintended and limited DNA methylation that should be associated with the grafting with the S44-end2 rootstocks (Fig. S1).

**Table S1.** DNA methylation status in LUC scions grafted onto S44-end2 or LUC rootstocks and the corresponding status of the S44-end2 plant.

Plant tissue	Percentage of methylated cytosine residues^a^		
	−341 to −292^b^	−291 to −91^b^ (target region)	−90 to −41^b^
Leaves of scion of LUC/end2	0	4.7	2.0
Leaves of scion of LUC/LUC	1.0	1.4	0
Leaves and petioles of S44-end2	78	68	12

^a^ The percentage of methylated cytosine residues as provided by the bisulfite sequence results.

^b^ Numbers indicate nucleotide position from TSP.

With respect to NPBTs, RNA silencing can be applied as a breeding technique to provide GM rootstocks with a useful trait, such as insect and/or viral resistance as well as tolerance to abiotic stress. Here, our present results show that grafting onto rootstocks showing RdDM caused slight changes in the transcriptomic and metabolomic profiles of non-GM scions. However, at present, we cannot find a causal relationship between these metabolites and DEGs. In addition, before linking these unintended changes in the omics profiles to safety concerns, we must also recognize that grafting itself often induces extensive changes in DNA methylation, especially in cases where grafting occurs between different species^47^^)^. Grafting-induced changes in DNA methylation are observed mainly within transposons and exon regions, and the mobile siRNA profile could account for such changes^48^^)^. Moreover, because grafting between plants of different species within the same family (or even from a different family) is currently practiced, we already consume foods that are epigenetically modified by grafting-induced changes in DNA methylation.

## 5. Conclusion

We have explored the possibility whether unanticipated transcriptomic, proteomic, and metabolomic changes in tobacco might have been exerted in non-GM scions engrafted with RdDM-inducing GM rootstocks. Only limited DNA methylation of target sequences was observed, and siRNAs harboring the CaMV 35S promoter sequences were detected at nearly negligible levels in the recipient scions. We found that expression profiles of several genes (DEGs, **Table S2**) and metabolites (**Fig. S2**) slightly changed in siRNA-recipient scions. However, the list of DEGs did not suggest a causal relationship between unknown metabolites and possible metabolic processes. Intensive epigenetic changes have already been induced in commercialized grafted plants, especially in those plants that have been subjected to grafting between plants of different species. We must carefully take such intensive RdDM in the conventional foods into consideration when the risk of scions grafted onto RdDM-inducible rootstocks is evaluated.

**Table S2.** List of functionally annotated DEGs between NN and NT.

**DEGs with Uniprot Annotation^a^**	**Accession**	**logFC^b^**
HMGL_IPONI HMG1/2-like protein	P40619	7.96
TMK3_ARATH Receptor-like kinase TMK3	Q9SIT1	6.85
ODPB1_ORYSJ Pyruvate dehydrogenase E1 component subunit beta-1	Q6Z1G7	6.16
* BT4_ARATH BTB/POZ and TAZ domain-containing protein 4	Q9FJX5	6.14
ATL3_ARATH RING-H2 finger protein ATL3	Q9XF63	6.14
YAC4_SCHPO Putative general negative regulator of transcription C16C9.04c	Q09818	6.11
KN7O_ARATH Kinesin-like protein KIN-7O	F4J2K4	6.07
ARAD1_ARATH Probable arabinosyltransferase ARAD1	Q6DBG8	6.03
* P2A13_ARATH F-box protein PP2-A13	Q9LEX0	5.86
1A110_ARATH Probable aminotransferase ACS10	Q9LQ10	5.75
GTE11_ARATH Transcription factor GTE11	Q93ZB7	3.74
STC_RICCO Sugar carrier protein C	Q41144	3.15
XYLT_ARATH Beta-1,2-xylosyltransferase	Q9LDH0	2.98
* TIF6B_ARATH Protein TIFY 6B	Q9LVI4	2.87
EIX2_SOLLC Receptor-like protein EIX2	Q6JN46	2.56
XTH8_ARATH Probable xyloglucan endotransglucosylase/hydrolase protein 8	Q8L9A9	2.30
OPT5_ARATH Oligopeptide transporter 5	Q9SUA4	2.20
Y5566_ARATH Uncharacterized protein At5g65660	Q9LSK9	2.10
SLAH3_ARATH S-type anion channel SLAH3	Q9FLV9	2.06
E13H_TOBAC Glucan endo-1,3-beta-glucosidase, acidic isoform PR-Q’	P36401	1.91
TSJT1_TOBAC Stem-specific protein TSJT1	P24805	1.63
PIRL4_ARATH Plant intracellular Ras-group-related LRR protein 4	Q9SVW8	1.63
SNAK2_SOLTU Snakin-2	Q93X17	1.23
CLH2_ORYSJ Clathrin heavy chain 2	Q2QYW2	1.04
TBB1_SOLTU Tubulin beta-1 chain	P46263	0.58
TOP2_PEA DNA topoisomerase 2	O24308	-1.03
UBIQP_HORVU Polyubiquitin (Fragment)	P0CG83	-1.09
ENL1_ARATH Early nodulin-like protein 1	Q9SK27	-1.13
THOC3_ARATH THO complex subunit 3	Q9FKT5	-1.18
SY111_ARATH Syntaxin-related protein KNOLLE	Q42374	-1.20
AUR1_ARATH Serine/threonine-protein kinase Aurora-1	Q9M077	-1.22
CDC2D_ANTMA Cell division control protein 2 homolog D	Q38775	-1.32
TGRM1_MOUSE TOG array regulator of axonemal microtubules protein 1	Q6A070	-1.37
CCN1_ANTMA G2/mitotic-specific cyclin-1	P34800	-1.53
HMG13_ARATH High mobility group B protein 13	Q9T012	-1.57
TOP2_PEA DNA topoisomerase 2	O24308	-1.63
CCN1_ANTMA G2/mitotic-specific cyclin-1	P34800	-1.68
PANS1_ARATH Protein PATRONUS 1	Q9LJG6	-1.75
TSNAX_MACFA Translin-associated protein X	Q4R599	-1.78
CTDSL_CHICK CTD small phosphatase-like protein	Q9PTJ6	-1.83
USPAL_ARATH Universal stress protein A-like protein	Q8LGG8	-1.98
NOP13_YEAST Nucleolar protein 13	P53883	-2.39
GPDA2_ARATH Glycerol-3-phosphate dehydrogenase [NAD(+)] 2	Q949Q0	-2.44
TRXM_BRANA Thioredoxin M-type	Q9XGS0	-2.53
PPME1_BOVIN Protein phosphatase methylesterase 1	Q58DN4	-2.81
PNSB3_ARATH Photosynthetic NDH subunit of subcomplex B 3	Q9LU21	-2.82
EPN1_ARATH Clathrin interactor EPSIN 1	Q8VY07	-3.39
RH7_SPIOL DEAD-box ATP-dependent RNA helicase 7	Q41382	-4.35
UBC2_WHEAT Ubiquitin-conjugating enzyme E2 2	P25866	-5.35
PLPHP_MOUSE Pyridoxal phosphate homeostasis protein	Q9Z2Y8	-5.59
1A110_ARATH Probable aminotransferase ACS10	Q9LQ10	-5.73
NFYA9_ARATH Nuclear transcription factor Y subunit A-9	Q945M9	-5.85
PSBP2_TOBAC Oxygen-evolving enhancer protein 2-2	P18212	-6.04
ZFP4_ARATH Zinc finger protein 4	Q39263	-6.70
CH20_ARATH 20 kDa chaperonin	O65282	-8.12

^a^ DEGs were identified P_FDR_ < 0.05.

^b^ Positive LogFC values indicate upregulated gene expression in NT relative to NN.

*Asterisk indicates the gene is functionally categorized as "response to wounding."

Kodama *et al.*Supplementary Table S2

**Table S3.**
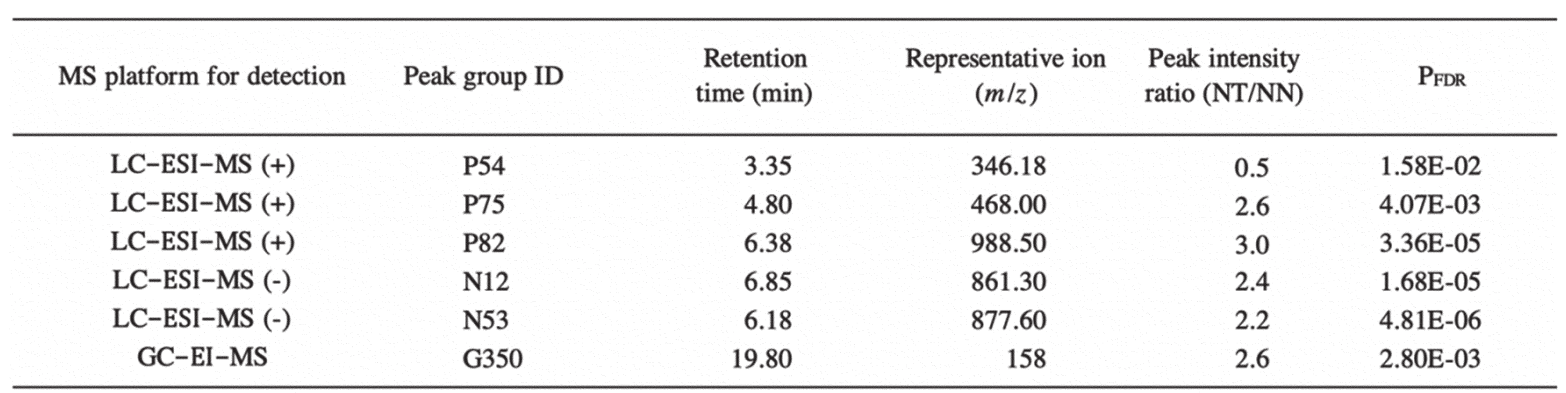
List of metabolite signals that showed more than twofold difference in abundance between NT and NN plants with P_FDR_ < 0.05.

In this study, we conducted a comprehensive analysis using transcriptomics, proteomics, and metabolomics to determine if any unexpected trait changes occurred. Such multi-omics analysis is an essential approach to comprehensively assess the content and composition of metabolites and allergenic substances that may be affected under fluctuating growth environments, various biotic and abiotic stresses, and epigenetic influences. In addition to such multi-omics approach, an important aspect for food safety assessment is the human food experience. Humans have cultivated various crop varieties in different environments and used them for food. During this long history, it has become clear that some species-specific substances require particular attention as food components. In other words, human food experience indicates the presence or absence of potentially harmful traits (e.g., alkaloids such as tomatine in tomato) that are not apparent in eating parts but may accumulate under uncommon conditions. Therefore, in addition to multi-omics analysis, detailed analyses based on eating experience are also to be conducted for food safety assessment.

## Acknowledements

This research was commissioned under the “2010 FSCJ Research Grant Program for Risk Assessment on Food Safety (Topic No. 1902).

## References

[1] Bradley S,Garner RJ. The grafter’s handbook: Revised & updated edition. Wilson J, ed. London. UK: Octopus Publishing Group, 2013. ISBN: 9781784724061.

[2] Melnyk CW,Meyerowitz EM. Plant grafting. *Current Biol*. 2015; **25**(5): R183–R188. PMID:25734263 doi:10.1016/j.cub.2015.01.029 10.1016/j.cub.2015.01.029 25734263

[3] Goldschmidt EE. Plant grafting: new mechanisms, evolutionary implications. *Front Plant Sci*. 2014; **5**: 727. PMID:25566298 doi:10.3389/fpls.2014.00727 10.3389/fpls.2014.00727 PMC4269114 25566298

[4] Koornneef M,Alonso-Blanco C,Peeters AJM,Soppe W. Genetic control of flowering time in Arabidopsis. *Annual Review of Plant Physiology and Plant Molecular Biology*. 1998; **49**(1): 345–370. PMID:15012238 doi:10.1146/annurev.arplant.49.1.345 10.1146/annurev.arplant.49.1.345 15012238

[5] Kobayashi Y,Kaya H,Goto K,Iwabuchi M,Araki T. A pair of related genes with antagonistic roles in mediating flowering signals. *Science*. 1999; **286**(5446): 1960–1962. PMID:10583960 doi:10.1126/science.286.5446.1960 10.1126/science.286.5446.1960 10583960

[6] Zeevaart JAD. Florigen coming of age after 70 years. *Plant Cell*. 2006; **18**(8): 1783–1789. PMID:16905662 doi:10.1105/tpc.106.043513 10.1105/tpc.106.043513 PMC1533981 16905662

[7] Guo Q,Liu Q,Smith NA,Liang G,Wang M-B. RNA silencing in plants: mechanisms, technologies and applications in horticultural crops. *Curr Genomics.* 2016; **17**(6): 476–489. PMID:28217004 doi:10.2174/1389202917666160520103117 10.2174/1389202917666160520103117 PMC5108043 28217004

[8] Palauqui JC,Elmayan T,Pollien JM,Vaucheret H. Systemic acquired silencing: transgene-specific post-transcriptional silencing is transmitted by grafting from silenced stocks to non-silenced scions. *EMBO J*. 1997; **16**(15): 4738–4745. PMID:9303318 doi:10.1093/emboj/16.15.4738 10.1093/emboj/16.15.4738 PMC1170100 9303318

[9] Stegemann S,Bock R. Exchange of genetic material between cells in plant tissue grafts. *Science*. 2009; **324**(5927): 649–651. PMID:19407205 doi:10.1126/science.1170397 10.1126/science.1170397 19407205

[10] Vaucheret H,Béclin C,Fagard M. Post-transcriptional gene silencing in plants. *J Cell Sci*. 2001; **114**(17): 3083–3091. PMID:11590235 doi:10.1242/jcs.114.17.3083 10.1242/jcs.114.17.3083 11590235

[11] Kehr J,Buhtz A. Long distance transport and movement of RNA through the phloem. *Journal of Experimental Botany*. 2007; **59**(1): 85–92. PMID:17905731 doi:10.1093/jxb/erm176 10.1093/jxb/erm176 17905731

[12] McGarry RC,Kragler F. Phloem-mobile signals affecting flowers: applications for crop breeding. *Trends Plant Sci*. 2013; **18**(4): 198–206. PMID:23395308 doi:10.1016/j.tplants.2013.01.004 10.1016/j.tplants.2013.01.004 23395308

[13] Notaguchi M. Identification of phloem-mobile mRNA. *Journal of Plant Research*. 2015; **128**(1): 27–35. PMID:25516498 doi:10.1007/s10265-014-0675-6 10.1007/s10265-014-0675-6 25516498

[14] Thomas HR,Frank MH. Connecting the pieces: uncovering the molecular basis for long‐distance communication through plant grafting. *New Phytologist*. 2019; **223**(2): 582–589. PMID:30834529 doi:10.1111/nph.15772 10.1111/nph.15772 30834529

[15] ISAAA Brief 53: Global Status of Commercialized Biotech/GM Crops: 2017. https://www.isaaa.org/resources/publications/briefs/53/default.asp.

[16] Zhao D,Song G. Rootstock-to-scion transfer of transgene-derived small interfering RNAs and their effect on virus resistance in nontransgenic sweet cherry. *Plant Biotechnol J*. 2014; **12**(9): 1319–1328. PMID:25132092 doi:10.1111/pbi.12243 10.1111/pbi.12243 25132092

[17] Kasai A,Bai S,Hojo H,Harada T. Epigenome editing of potato by grafting using transgenic tobacco as siRNA donor. *PLoS ONE*. 2016; **11**(8): e0161729. PMID:27564864 doi:10.1371/journal.pone.0161729 10.1371/journal.pone.0161729 PMC5001710 27564864

[18] Yoo BC,Kragler F,Varkonyi-Gasic E,et al. A systemic small RNA signaling system in plants. *Plant Cell*. 2004; **16**(8): 1979–2000. PMID:15258266 doi:10.1105/tpc.104.023614 10.1105/tpc.104.023614 PMC519190 15258266

[19] Shaharuddin NA,Han Y,Li H,Grierson D. The mechanism of graft transmission of sense and antisense gene silencing in tomato plants. *FEBS Letters*. 2006; **580**(28-29): 6579–6586. PMID:17113082 doi:10.1016/j.febslet.2006.11.005 10.1016/j.febslet.2006.11.005 17113082

[20] Kasai A,Bai S,Li T,Harada T. Graft-transmitted siRNA signal from the root induces visual manifestation of endogenous post-transcriptional gene silencing in the scion. *PLoS ONE*. 2011; **6**(2): e16895. PMID:21347381 doi:10.1371/journal.pone.0016895 10.1371/journal.pone.0016895 PMC3036722 21347381

[21] Md Ali E,Kobayashi K,Yamaoka N,Ishikawa M,Nishiguchi M. Graft transmission of RNA silencing to non-transgenic scions for conferring virus resistance in tobacco. *PLoS One.* 2013; **8**(5): e63257. PMID:23717405 doi:10.1371/journal.pone.0063257 10.1371/journal.pone.0063257 PMC3661558 23717405

[22] Melnyk CW,Schuster C,Leyser O,Meyerowitz EM. A developmental framework for graft formation and vascular reconnection in *Arabidopsis thaliana*. *Current Biol*. 2015; **25**(10): 1306–1318. PMID:25891401 doi:10.1016/j.cub.2015.03.032 10.1016/j.cub.2015.03.032 PMC4798781 25891401

[23] Podevin N,Devos Y,Davies HV,Nielsen KM. Transgenic or not? No simple answer! New biotechnology-based plant breeding techniques and the regulatory landscape. *EMBO Rep*. 2012; **13**(12): 1057–1061. PMID:23154464 doi:10.1038/embor.2012.168 10.1038/embor.2012.168 PMC3512411 23154464

[24] Kodama H,Miyahara T,Oguchi T,et al. Effect of transgenic rootstock grafting on the omics profiles in tomato. *Food Saf (Tokyo)*. 2021; **9**(2): 32–47. PMID:34249588 doi:10.14252/foodsafetyfscj.D-20-00032 10.14252/foodsafetyfscj.D-20-00032 PMC8254850 34249588

[25] Hirai S,Takahashi K,Abiko T,Kodama H. Loss of sense transgene-induced post-transcriptional gene silencing by sequential introduction of the same transgene sequences in tobacco. *FEBS J*. 2010; **277**(7): 1695–1703. PMID:20180844 doi:10.1111/j.1742-4658.2010.07591.x 10.1111/j.1742-4658.2010.07591.x 20180844

[26] Tomita R,Hamada T,Horiguchi G,Iba K,Kodama H. Transgene overexpression with cognate small interfering RNA in tobacco. *FEBS Letters*. 2004; **573**(1-3): 117–120. PMID:15327985 doi:10.1016/j.febslet.2004.07.063 10.1016/j.febslet.2004.07.063 15327985

[27] Kodama H,Hamada T,Horiguchi G,Nishimura M,Iba K. Genetic enhancement of cold tolerance by expression of a gene for chloroplast [omega]-3 fatty acid desaturase in transgenic tobacco. *Plant Physiol*. 1994; **105**(2): 601–605. PMID:12232227 doi:10.1104/pp.105.2.601 10.1104/pp.105.2.601 PMC159399 12232227

[28] Nishiuchi T,Nakamura T,Abe T,Kodama H,Nishimura M,Iba K. Tissue-specific and light-responsive regulation of the promoter region of the *Arabidopsis thaliana* chloroplast omega-3 fatty acid desaturase gene (FAD7). *Plant Mol Biol*. 1995; **29**(3): 599–609. PMID:8534855 doi:10.1007/BF00020987 10.1007/BF00020987 8534855

[29] Edwards K,Johnstone C,Thompson C. A simple and rapid method for the preparation of plant genomic DNA for PCR analysis. *Nucleic Acids Res*. 1991; **19**(6): 1349. PMID:2030957 doi:10.1093/nar/19.6.1349 10.1093/nar/19.6.1349 PMC333874 2030957

[30] Iijima Y,Nakamura Y,Ogata Y,et al. Metabolite annotations based on the integration of mass spectral information. *Plant J*. 2008; **54**(5): 949–962. PMID:18266924 doi:10.1111/j.1365-313X.2008.03434.x 10.1111/j.1365-313X.2008.03434.x PMC2440531 18266924

[r31] 31.Holman JD, Tabb DL, Mallick P. Employing ProteoWizard to convert raw mass spectrometry data. *Curr Protoc Bioinformatics*. 2014; 46 (1): 13.24.1–13.24.9. PMID: 24939128, doi:10.1002/0471250953.bi1324s46. 10.1002/0471250953.bi1324s46 PMC4113728 24939128

[32] Tautenhahn R,Patti GJ,Rinehart D,Siuzdak G. XCMS Online: a web-based platform to process untargeted metabolomic data. *Analytical Chemistry*. 2012; **84**(11): 5035–5039. PMID:22533540 doi:10.1021/ac300698c 10.1021/ac300698c PMC3703953 22533540

[33] Ogawa T,Kashima K,Yuki Y,et al. Seed metabolome analysis of a transgenic rice line expressing cholera toxin B-subunit. *Scientific Reports*. 2017; **7**(1): 5196. PMID:28701756 doi:10.1038/s41598-017-04701-w 10.1038/s41598-017-04701-w PMC5507873 28701756

[34] De Vos RCH,Moco S,Lommen A,Keurentjes JJB,Bino RJ,Hall RD. Untargeted large-scale plant metabolomics using liquid chromatography coupled to mass spectrometry. *Nature Protocols*. 2007; **2**(4): 778–791. PMID:17446877 doi:10.1038/nprot.2007.95 10.1038/nprot.2007.95 17446877

[35] Tsugawa H,Tsujimoto Y,Arita M,Bamba T,Fukusaki E. GC/MS based metabolomics: development of a data mining system for metabolite identification by using soft independent modeling of class analogy (SIMCA). *BMC Bioinformatics*. 2011; **12**(1): 131. PMID:21542920 doi:10.1186/1471-2105-12-131 10.1186/1471-2105-12-131 PMC3102042 21542920

[36] Chong J,Soufan O,Li C,et al. MetaboAnalyst 4.0: towards more transparent and integrative metabolomics analysis. *Nucleic Acids Res*. 2018; **46** (W1): W486–W494. PMID:29762782 doi:10.1093/nar/gky310 10.1093/nar/gky310 PMC6030889 29762782

[37] Sijen T,Vijn I,Rebocho A,et al. Transcriptional and posttranscriptional gene silencing are mechanistically related. *Curr Biol*. 2001; **11**(6): 436–440. PMID:11301254 doi:10.1016/S0960-9822(01)00116-6 10.1016/s0960-9822(01)00116-6 11301254

[38] Okano Y,Miki D,Shimamoto K. Small interfering RNA (siRNA) targeting of endogenous promoters induces DNA methylation, but not necessarily gene silencing, in rice. *Plant J*. 2008; **53**(1): 65–77. PMID:17971040 doi:10.1111/j.1365-313X.2007.03313.x 10.1111/j.1365-313X.2007.03313.x 17971040

[39] Hirai S,Kodama H. RNAi vectors for manipulation of gene expression in higher plants. *The Open Plant Science Journal*. 2008; **2**(1): 21–30. doi:10.2174/1874294700801010021

[40] Voinnet O,Vain P,Angell S,Baulcombe DC. Systemic spread of sequence-specific transgene RNA degradation in plants is initiated by localized introduction of ectopic promoterless DNA. *Cell*. 1998; **95**(2): 177–187. PMID:9790525 doi:10.1016/S0092-8674(00)81749-3 10.1016/s0092-8674(00)81749-3 9790525

[41] Molnar A,Melnyk CW,Bassett A,Hardcastle TJ,Dunn R,Baulcombe DC. Small silencing RNAs in plants are mobile and direct epigenetic modification in recipient cells. *Science*. 2010; **328**(5980): 872–875. PMID:20413459 doi:10.1126/science.1187959 10.1126/science.1187959 20413459

[42] Shimamura K,Oka S,Shimotori Y,Ohmori T,Kodama H. Generation of secondary small interfering RNA in cell-autonomous and non-cell autonomous RNA silencing in tobacco. *Plant Mol Biol*. 2007; **63**(6): 803–813. PMID:17225952 doi:10.1007/s11103-006-9124-9 10.1007/s11103-006-9124-9 17225952

[43] Li S,Wang X,Xu W,et al. Unidirectional movement of small RNAs from shoots to roots in interspecific heterografts. *Nat Plants*. 2021; **7**(1): 50–59. PMID:33452489 doi:10.1038/s41477-020-00829-2 10.1038/s41477-020-00829-2 33452489

[44] Tamiru M,Hardcastle TJ,Lewsey MG. Regulation of genome-wide DNA methylation by mobile small RNAs. *New Phytologist*. 2018; **217**(2): 540–546. PMID:29105762 doi:10.1111/nph.14874 10.1111/nph.14874 29105762

[45] Nakamura S,Hondo K,Kawara T,et al. Conferring high-temperature tolerance to nontransgenic tomato scions using graft transmission of RNA silencing of the fatty acid desaturase gene. *Plant Biotechnol J*. 2016; **14**(2): 783–790. PMID:26132723 doi:10.1111/pbi.12429 10.1111/pbi.12429 PMC11389092 26132723

[46] Kodama H,Iwasa H,Hirai S,Oka S. Amplification of small interfering RNAs in transgenic plants. Agriculture Research and Technology. Bundgaard K, Isaksen L, eds. New York, USA: Nova Science Publishers; 2010: 379–395.

[47] Tsaballa A,Xanthopoulou A,Madesis P,Tsaftaris A,Nianiou-Obeidat I. Vegetable grafting from a molecular point of view: the involvement of epigenetics in rootstock-scion interactions. *Front Plant Sci*. 2021; **11**: 621999. PMID:33488662 doi:10.3389/fpls.2020.621999 10.3389/fpls.2020.621999 PMC7817540 33488662

[48] Cao L,Yu N,Li J,Qi Z,Wang D,Chen L. Heritability and reversibility of DNA methylation induced by in vitro grafting between *Brassica juncea* and *B. oleracea*. *Scientific Reports*. 2016; **6**(1): 27233. PMID:27257143 doi:10.1038/srep27233 10.1038/srep27233 PMC4891673 27257143

